# Ultrasound and Sonogenetics: A New Perspective for Controlling Cells with Sound

**DOI:** 10.22037/ijpr.2021.114868.15079

**Published:** 2021

**Authors:** Seyedeh Sara Azadeh, Parinaz Lordifard, Mohammad Hasan Soheilifar, Gholamreza Esmaeeli Djavid, Hoda Keshmiri Neghab

**Affiliations:** a *Department of Biology, Sciences and Research Branch, Islamic Azad University, Tehran, Iran. *; b *Institute of Biochemistry and Biophysics, University of Tehran, Tehran, Iran. *; c *Department of Medical Laser, Medical Laser Research Center, Yara Institute, ACECR, Tehran, Iran. *; d *Department of Photo Healing and Regeneration, Medical Laser Research Center, Yara Institute, ACECR, Tehran, Iran.*

**Keywords:** Ultrasound, Sonogenetics, Mechano-sensitive proteins, Imaging, Sound-sensitive proteins

## Abstract

An important challenge in neurobiology is to stimulate a single neuron, especially in deep areas of the brain. The optogenetics methods need a surgical operation to convey light sources to targeted cells. Nowadays, non-invasive tools such as sonogenetics with the ability to modulate and visualizing cellular and molecular processes have attracted much attention. The study of the biological functions of living organisms always requires tools for monitoring and imaging dynamically. Current sonogenetic approaches use ultrasound as a non-invasive tool to precisely control cellular function. In general, sonogenetics includes the development of mechano-sensitive proteins, approaches for introducing their genes to specific cells, targeted stimulation, and finally, reading the outcome. Hence, to prepare a short review of emerging technology sonogenetics, we summarized the introduction of sound waves, the mechano-sensitive proteins commonly used in sonogenetics, and potential therapeutic applications of sonogenetics for biological research and medicine. This short review would beneficiate in the translation of sonogenetics from present *in-vitro *and *in-vivo *investigations to clinical therapies.

## Introduction

Today sound and light are known as fast transfer tools in non-invasive therapeutic methods such as sonogenetics and optogenetics. Therefore, a study in this field can be useful for imaging and modulating biological systems. Sound waves are known as the strongest mechanical waves that are divided into three types of sound; 1) Audible sound with the frequency of 20 Hz to 20 kHz is perceived by the auditory organ and the nervous system. Audible sound enters the human ears as mechanical vibration, then converts to biomechanical signal by inner ear hair cells and transmitted to the brain as a neuronal signal, 2) Infrasound wave with a frequency of less than 20 Hz is inaudible for the human’s ear. These waves were first discovered in 1883 in Krakatao, Indonesia, and with low frequency, they can travel over thousands of kilometers. Infrasound is not used in therapy but is very important in biological phenomena and geology (1). 3) Another type of sound wave is ultrasound with a frequency above 20 kHz which has been very important in diagnosis. The first use of ultrasound was as a diagnostic tool for brain tumors, which was done by Theodore and Frederick Dussik in the 1930s (2). Ultrasound was first discovered in 1876 by Francis Galton, who made the radar-making device on World War 1 submarines (3). Nowadays, the use of ultrasound is one of the most common clinical applications in diagnosis and treatment, especially in the field of neuroscience. 

**Figure 1:**
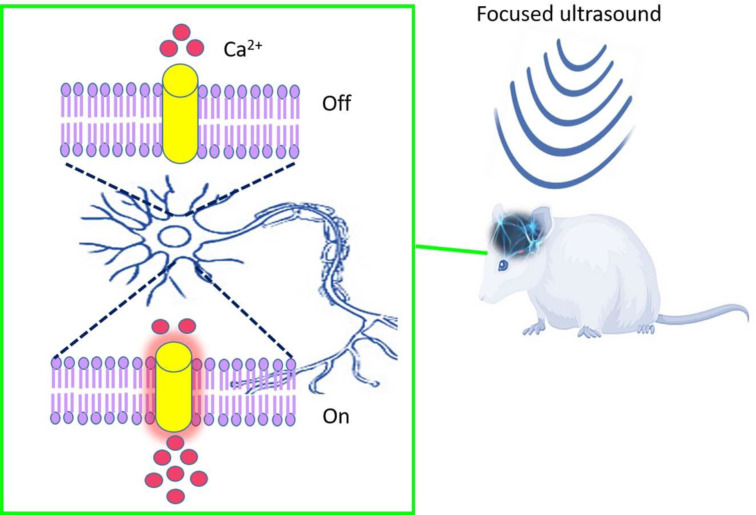
Schematic representation of focused ultrasound for neuromodulation in rodent brain

Ultrasound is the most efficient tool in physiotherapy, surgical instruments, chemotherapy, drug delivery, sonography, and the high-intensity focused ultrasound (HIFU) field (4). Notably, ultrasound waves used in sonography in the frequency range of 2 to 18 MHz, are a hundred times greater than the threshold of human hearing. In ultrasound waves, higher frequencies have smaller wavelengths and higher energy and finally could penetrate soft tissues. The penetration depth of sound waves is the most distinguishable factor between sonogenetics and optogenetics. Although, optogenetics technology provides helpful tools to study biological systems, it may be less efficient in deep tissues (5). Ultrasound, like any wave, also has speed in soft tissue nearly 1540 m/s. The acoustic impedance of ultrasound becomes noticeable at the boundary of various media. Note that there is no significant difference between the acoustic impedances for soft tissue, and that’s why the sound wave penetrates homogeneously in the soft tissue and provides simple images without distortion (6). Gradually, a new therapeutic modality has emerged in the treatment of medical disorders by using ultrasonic energy to target regions in the body. Focused Ultrasound (FUS) permits sonic energy to be targeted in deep tissue, non-invasively, and precisely (Figure 1) (7, 8). 

Although the ultrasound mechanism on cellular excitability is poorly understood, there are several possibilities of energy delivery of ultrasound being considered; firstly, heat production by ongoing ultrasound waves can be used for thermal bioswitches. Secondly, cavitation occurs via the interaction of ultrasound with microbubbles, which can cause cell or vascular obstacle infraction. Ultrasounds also deposit momentum when they travel through the medium, produced by mechanical forces called acoustic radiation forces that are able to stimulate sound-activated molecules (9).

The currently available tool for monitoring and controlling cell events is optogenetics. Since each technique is not perfect and optogenetics is not an exception to this rule, light scattering has been difficult to use this technique in biological systems. In contrast, ultrasound has a long history in biomedical imaging and therapeutics, but its application in manipulating and monitoring cellular events is limited. Recent progress has been started to solve this restriction by the discovery of ultrasound-responsive elements that allow ultrasound to link to the cell activities directly, in a new technology called “sonogenetics”. Sonogenetics, which utilizes ultrasound to noninvasively manipulate and control cells genetically engineered with ultrasound-responsive proteins, can be widely applied to manipulate cellular functions. This investigation aims to introduce this emerging technology and provide a clue for further studies. Additionally, this study has been centralized on the latest developments in ultrasound and specially sonogenetics technology in medicine. Four basic steps are required for sonogenetics which are briefly discussed below.

*Part 1: Sound-sensitive proteins*

One of the main challenges in the development of non-invasive technologies such as sonogenetics is to find the appropriate sound-sensitive proteins as toolboxes. Since sonogenetics used mechanical ultrasound waves, the mechano-sensitive ion channels genes have been identified and expressed in the target cells. Different types of mechano-sensitive channels genes act in response to different mechanical stimuli containing shear stress, osmotic pressure, stretch, and pressure. Mechanical forces applied to mechano-sensitive channels lead to bilayer deformation and movement of the channel helix and finally, opening the pore, flux of the ions, and small molecules.

Recent studies have been proposed mechano-sensitive ion channels include MEC-4 in *Caenorhabditisel elgans* (10), TREK-1, TREK-2 (9), MSCL (11), Piezo1 (12, 13) ,transient receptor potential such as human TRPA-1 (14), TRP-4 (15),TRPV-1 (15) and TRAAK K+ (15) which are main actuators in sonogenetics (Table 1). 

In addition to the above, another type of sound-sensitive protein in the mouse brain is called Prestin and can be stimulated by ultrasound (16). The effects of ultrasound on targeted tissues are through the mechanical and thermal mechanisms. Transient receptor potential vanilloid 1 (TRPV-1) is one of the thermo-sensitive ion channels that are used in sonogenetic and activated at 42 °C (17). The TRPV-1 channels closed at the physiological temperature of the body to keep the cells safe. These channels are one of the primary proteins that are used in sonogenetics and expressed in non-neural cells and nerve fiber such as muscle cells and vascular endothelial cells (18, 19). Protein kinase C and phospholipase C as intracellular signaling pathway elements have a regulatory effect on TRPV1 activity. The phosphatidyl inositol-bisphosphate (PIP2) also has an inhibitory effect on this channel (20). The activation mechanism of this channel is a result of some factors such as heat (above 42 °C), acidic environment, and chemical stimuli, including capsaicin (which is abundant in red pepper) (21). The transient receptor potential Ankyrin1 (TRPA-1), like TRPV-1, is a subgroup of TRP channels that permeable to calcium. The agonist compound of TRPA-1 is mustard oil that is activated by the allylisothiocyanate component. Some compounds as toxic scorpion peptides (WaTx) and electrophilic irritants can be activating by TRPA-1 channels. The structure of TRPA-1 includes six transmembrane domains throughout the plasma membrane, 16 ankyrin, intracellular N-terminal and C-terminal domains. This region of TRPA-1 has an important role in mechano-sensitive features in this channel (22). Notably, TRPA-1 channels were activated at cold temperature (<17 °C) (23). One type of TRPA-1 in humans (hsTRPA-1) is used as a sonogenetic toolbox. Researchers found that following ultrasound simulation on this channel, the intracellular calcium level, and membrane potential are increased (22). Other mechano-sensitive proteins in bacteria are MS channels. These channels found in bacteria such as *E.coli* are divided into different subgroups such as; MscL (mechano-sensitive channel large), MscS (mechano-sensitive channel small,) and MscM (mechano-sensitive channel mini) (24). The MscS family members are found widely among bacteria and archaea. Moreover, they are found in all of the plant’s genome, fungi and eukaryotes genome.

MscL protein consists of a polypeptide with 136 residues of amino acid and signals recognition particle (SRP) to target in the plasma membrane (25) and two alpha-helices in both sides of the plasma membrane (26) Some factors such as light and pH can affect Mscl (27, 28). Ultrasound is an important factor to open the MscL and control target cell activities. The frequency of ultrasound waves can improve the target cell activity by rapid chemical interaction. K2P family channels are new protein channels of potassium with two domains, four transmembrane domains, and extracellular caps. TREK-1, TREK-2, and TRAAK are mechano-sensitive channels of the K2P family that being activated by mechanical force. The genes of these channels are expressed in the nervous system of mammalian and have efficient effects on vital signs. The activation of these channels is strongly dependent on the extracellular K^+^ level (29). Prestin, one of the transmembrane proteins of the cochlea, is a voltage-to-force transformer motor. All changes and movements in the outer hair cell (OHC) of cochlea make a membrane potential in these cells and that might be the cause of auditory messages in mammalian. Prestin has a high sensitivity to the frequency of sound in hearing organs of mammals (30) and can detect a sound frequency in the range of less than 20 kHz. Prestin function depends on electromechanical signals send from the OHC of the cochlea to the brain (30). It is noteworthy that prestin isn’t an ion channel and does not have any role in ion exchange on either side of the membrane (15).

*Part 2: Gene delivery and expression*

Gene coding ultrasound-sensitive protein can be delivered to the target cells via gene delivery. This process is done with viral-vectors and non-viral vectors methods and also the creation of transgenic lines.

*Part 3: Ultrasound exposure*

As mentioned above, ultrasound is an acoustic wave with a frequency of more than 20000 Hz. Focused high-intensity ultrasound is a non-invasive surgical technique that uses focused ultrasound for thermal ablation of tissues. Focused ultrasound exerts its effect with the most appropriate energy and minimum period time (31). To have a better effect and more efficiency of ultrasound radiation, some parameters such as intensity, fundamental frequency, duration, duty cycles, and pulse repetition frequency are checked. 

Intensity: intensity is the amount of acoustic energy produced by ultrasound that is described as Spatial-peak Pulse-average (Isppa) or spatial-peak time-average (Ispta) that can be used for the safety radiation of ultrasound in brain simulation. Focused ultrasound waves are divided into two types based on intensity: Focused ultrasound with high intensity (HIFU) and Focused ultrasound with low intensity (LIFU). The intensity of HIFU is approximately about 100 w/cm^2^ to 10 kw/cm^2^ and due to its high intensity; it’s widely used in treatment and surgery (32). Sometimes high intensity can cause an increase in the temperature and make irreversible destructive effects. LIFU with an intensity less than 3 w/cm^2^ can modulate the local tissue by controlled temperature. Ultrasound stimulation can record electrical activity when its intensity increases up to 100 mw/cm^2^ and after reaching 100 mw/cm^2^, it is impossible to analyze brain activity (33). 

Fundamental frequency: The oscillation cycle per time is called fundamental frequency which is widely used in ultrasound applications. Ultrasound has a wide frequency range but limited frequencies can be effective in diagnosis and treatment. For example, ultrasound with high frequency (1-20 MHz) uses in diagnosis and ultrasound with medium frequency (0.7-3MHz) utilizes in therapy and ultrasound with low frequency (20-200 kHz) uses in industry. Higher frequency waves have smaller depth penetration. Therefore, for high penetration depth, low frequencies are essential (34).

Duration: The interval between the duration of pulse transmission. Researchers have found that long-term (>10 s) use of LIFU can inhibit neural activities while using short-term can be simulated (35).

Pulse repetition frequency (PRF): PRF is the number of ultrasound pulses over a specified period of time that is typically measured as hertz (Hz) or cycle per second. Recent studies have shown that PRF levels are associated with neuron modulation. PRF above 500 Hz of ultrasound simulates neural activity with evoked EEG (36).

Duty cycle (DC): the ratio of the ultrasound cycle per pulse is called the duty cycle (DC). The duty cycle of ultrasound can be delivered in the continuous or discrete pulse, which is DC = 100% if the ultrasound is continuous and without any interruption. Noteworthy, in most studies pulsing duty cycle stimulation of ultrasound with DC < 100 can be more efficient in neural activation application (37).

*Part 4: Readout*

The results induced by stimulating the ultrasound-sensitive proteins require to be evaluated in cells, tissue or organisms. Electrodes and arrays can be used to record the effect of changes in membrane voltage by evaluation of calcium charges after ultrasound exposure. Many biosensors such as dye and genetically encoded indicators can be used to evaluate different cellular readouts. Ultimately, investigating cell behavior can be used to evaluate the effect of modulating cellular activity *in-vivo*.

**Application of ultrasound**

As mentioned above, ultrasound is a type of mechanical wave and can be focused on a high frequency. Sound waves travel among the tissues at 1540 m/s and scatter from the interface of tissues with different acoustic impedance which is a function of the density and compressibility. Ultrasound Imaging is a diagnostic modality in the clinic and there are numerous ultrasound imaging modes such as B-mode imaging, doppler imaging which detects the motion of the red blood cells, contrast imaging that relies on the administration of contrast agents like microbubbles, ultrafast imaging, functional ultrasound imaging, and ultrasound localization microscopy. Furthermore, ultrasound can interact with biomolecules to enhance their transport through cellular and tissues barrier which is relied on the cavitation behavior of microbubbles and leads to cellular sonoporation, vascular barrier opening, acoustic trapping, and manipulation of cells and molecules. Furthermore, it is worth mentioning that ultrasound can be combined with other forms of energy such as light and magnetic fields to enable the imaging or actuation of biomolecules that can be considered as photoacoustic imaging, acoustically modulated light focusing, and acoustically modulated magnetic resonance (6).

The physics of ultrasound makes it a favorable option for neuromodulation because it can be focused on millimeter resolutions through the skull bone to deep-brain regions.

Ultrasound waves associated with heat generation can be effective in therapy; In fact, they can be used in generating internal heating in local tissue without adverse effects. In recent studies, researchers have found a way to use ultrasound to treat a specific type of cancer by focused ultrasound. The focused ultrasound can upgrade the temperature of the area of the tumor without any destructive effect on the surrounding tissue.

Most medical diagnostic imaging procedures are performed with X-rays. X-ray photons have high energy and high ionizing radiation. Due to this feature, X-rays can break down molecular bonds in tissues. This demolition in molecular bonds can lead to a change in function or the destruction of tissues. Unlike X-ray, there is no ionizing radiation exposure related to ultrasound. So using the FUS is recommended in sensitive situations that X-rays can be dangerous. Besides, ultrasound can be discriminate contrast between different types of soft tissue. Ultrasound can participate in some biomedical applications. The study of using ultrasound in the treatment of cancer is a novel field for researchers. Surgery is the common method to treat solid tumors but this method is not applicable in some tumors in sensitive areas. Findings have shown that thermal ablation of the HIFU approach can also be used in curing cancers such as prostate cancer, breast cancer, liver cancer, and kidney cancer which is discussed below (38).

Prostate cancer: Prostate cancer is the most common cancer among men. HIFU is one of the appropriate choices for the treatment of prostate cancer. Since surgery can cause destruction effects on the function of urinary and sexual ducts, the use of this technique may be useful. The prostate is located in a deep area of the pelvis and ultrasound can detect the prostate with high accuracy and minimal damage for adjacent tissue (39).

Breast cancer: Breast cancer is the second most common cancer in females worldwide. This cancer is caused by inheritance, and environmental factors such as age; obesity, moreover, alcohol consumption increases the risk of breast cancer infection in women. The common methods in the treatment of breast cancer are surgery, chemotherapy, radiotherapy, and hormone therapy. FUS as a non-invasive tool appropriate method instead of mastectomy. In this method, HIFU directed into the tumor of tissue and terminated the tumors by increasing temperature without injury. Using HIFU to treat breast cancer can prevent the proliferation, invasion, and metastasis of breast cancer (40). 

Liver cancer: Surgery coupled with implant liver is the most promising way to cure (39). The liver has many blood vessels and because of that reason, chemotherapy and surgery is the main method for this cancer. Studies have shown that HIFU can destroy tumors completely in the selection area in the liver cancer with the least pain for patients. Doxorubicin administration combined with HIFU treatment increases the chance of survival of the patient (41).

Kidney cancer: Surgery is the main method to treat kidney cancer but since most kidneys tumors are small, a non-invasive method is the best treatment choice for this cancer, and for this aim, using HIFU is recommended.

**Sonogenetics and its applications**

Currently, LIFU is a non-surgical approach used for neuromodulation of the peripheral and central nervous systems. Although, significant advances have been done in neuromodulation, still faces some limitation such as lack of spatial selectivity. Sonogenetics has appeared as a novel strategy to target individual cells with high spatial resolution.

Comprehension of how the neural system works and how triggers particular behavior need recognition of participating neurons and their activities.

Some strategies have been advanced for manipulating neural circuits using small molecules (pharmacogenetics) (42) or light (optogenetics) (43, 44). While these approaches have unveiled some complications in neural circuits, they have some limitations; problems in the transfer of exciter to specific neurons in deep areas of the brain. To solve this matter a novel approach has been elaborated which genetically sensitizes selected neuron cells to ultrasound. Integration of deep penetration and spatial targeting of ultrasound has emerged in sonogentics technology that has the potential clinical application of Epilepsy, Depression, and Parkinson’s disease (45).

Besides, it is shown that HIFU can change neuronal activity in frog and turtle neuromuscular systems, but heating tissues by HIFU shows risks for irreversible damage; Thus, recent studies have focused on the use of Low-Intensity of Focused Ultrasound (LIFU).

Chalasani and his colleagues used ultrasound to stimulate specific neurons in the nematode, *Caenorhabditis Elegans* and observed behavioral responses to single ultrasound pulses depending on the pressure of the ultrasound, but it accrued just in the situation that tiny bubbles were added and surrounded the worm body that amplify the US and cause mechanical stimulus: because wild-type animals are insensitive to low-pressure ultrasound and require gas-filled microbubbles to transduce the ultrasound waves. But they found that the mechanosensory channel formed by TRP-4 sensitizes neurons to ultrasound stimulus and mediated the responses also in other animals called mutated animals (22). 

This new technique became the basis of some new technique applications. Therefore, using sonogenetics, activation of neurons does not require direct contact of TRP-4 expressing neurons with microbubbles, since internally localized neurons could be manipulated by this method and since ultrasound can penetrate the skull, it is so interesting to know if it is possible to use songenetics in other organisms or not (45).

Also, it is shown that LIFU with the frequency of 1.1 MHz and intensity of 14-93 w/cm^2 ^can activate the peripheral neuronal structures in the human hand and other surveys; ultrasound was used due to manipulating the structure of the deep neurons of the human hand to decrease the chronic pain (45).

These findings open doors to different applications of the effects of ultrasound on an existing or an engineered ultrasound-sensitive ion channel that could be over-expressed in a particular region in a cell carrying specific genetic markers (9).

In 2016, scientists found that focused ultrasound modulates K^+ ^current K2P channels and also Na^+ ^current of Na_5 _1.5 which are expressed in neurons, retinal cells or cardiac cells which may lead to important medical applications. Lots of the brain and the heart ion channels might respond to mechanical or temperature-related effects related to the US application (9). In addition to neuromodulation, sonogenetics could be applied to manipulate and control a variety of types of cells and tissues from cardiomyocytes pacemaker cells in the heart to insulin-secreting cells in the pancreas. However, additional investigations are needed to explore the therapeutic potential of this novel technology.

On the other side, sonogenetics has some other applications, for example, sonogenetics in fetal neurology, which incorporating the idea of fetuses first will play an important role in the molecular genetics era.

As we know, bacterial and eukaryotic cells may sense physiologically relevant changes in the membrane tension using MscL and MscS homologs, then convert tension into solute flow across the membrane and at last turn, these fluxes into applicable actions such as osmotic shock protection in some type of bacteria and some others like *B. subtilis*, MS channels have a crucial role in the process of exiting stationary phase and re-entering the growth cycle. 

Moreover, *Arabidopsis thaliana* encodes ten MscS-Like (MSCL) proteins in the plasma and vacuolar membranes that are regulated by salt and other osmotic (46).

In addition to the above, in a survey, researchers expressed MscL genes in rat hippocampal neurons in primary culture and activated it by low-pressure ultrasound pulses, because the gain of function mutation, 192L, had sensitized MscL to low-pressure ultrasound that can penetrate the skull and brain tissue with very little impedance or damage in tissue and triggering action potentials at a peak negative pressure. MscL can be activated in any membrane independent of other proteins or ligands and it has a single small gene that can easily be targeted to a specific neuron *in-vivo*. As a fact, the different or additional mutations can generate new sensors suited to different needs. For example, molecular engineering of MscL variants with fast gating kinetics which is combined with strong currents and also rapid inactivation can improve the frequency of ultrasound-evoked spikes or more accurate manipulation can be achieved by MscL modification with designed ion selectivity and pore size.

It is noteworthy to tell that ultrasound should be able to deliver drugs to the cells through installed MscL in the membrane called probably “sonotherapy” because scientists have used transgenic MscL to deliver phalloidin into mammalian cells.

MscS-like proteins also are implicated in cellular signal transduction pathways. Sonogenetics by the targeted use of different types of channels in special microorganisms, tissues, or cells can use their features to apply different acts in the way it did not work before or also change the channel’s activity to evaluate their activities. For example, examine the biochemical and biophysical consequence of osmotic shock, evaluation of the role played by MS channel in cellular processes that changes membrane tension other than osmotic stress like rehydration of bacteria and pollen spores, membrane remodeling during cell or organelle fission, changes in cell morphology or size, and altered membrane synthesis and neural circuit activity (6).

Moreover above, sonogenetics can have a crucial role in actuating the cellular signaling according to some of its features like ultrasound ability to apply mechanical forces to tissues in a controlled temperature increase that leads to mechanical actuation of receptors, ultrasound neuromodulation that would provide sonogenetics control of cellular function. Specific examples are the proliferation of microbes in the gut, the release of the cell expressing therapeutic payload, and the excitability of specific neurons (6).

A more recent study by He *et al.* designed a sonogentics nanosystem by expressing MSCL, mechanosensor ion channels, in tumor cells. Following ultrasound stimulation, MSCL-expressing cells are overloaded with Ca^2+^ fluxes and subsequently triggering cell apoptosis. So it is feasible to precisely control cell apoptosis without affecting the rest of the cells (47).

**Table 1:** Mechano-sensitive proteins in sonogenetics

**Mechano-sensitive protein family**	**Mechano-sensitive protein**	**character**	**Reference**
TRP	TRPV-1	activated at 42 °C	(17)
	TRPA-1	activated in cold temperature (<17 °C)	(23)
	TRP-4	Expressed in nociceptive neurons and increase cell sensitivity to ultrasound	(15)
Ms	MscL	Large conductance activated in the low pressure of US	(27, 11)
	MscS	Small conductance protects cells from lysis by releasing osmolytes.	(24, 11)
TRNs	MEC-4	Expressed in neurons and epithelium cells, activated by all simulations on the skin	(10)
K2P channels	TREEK-1	Expressed in the nervous system in mammalian, activation, and modulation of activity by increasing the concentration of extracellular potassium	(30)
	TREEK-2		
	TRAAK K+		

## Conclusion

Currently, optogenetics has known as a significant and useful tool in modulating and monitoring various cells, comprehension of different disorders and has been identified as an alternative to conventional electrical stimulation methods used in clinical research. Light penetration is a major factor limiting this valuable technology in deep area structures. The Sonogenetics approach is an alternative tool that provides a new strategy for deep tissue high resolution and non-invasive therapy. Sonogenetics might be the ideal manipulation technique for Neurostimulation like in the heart and brain compared to optogenetics. Furthermore, Sonogenetics have promising applications not only in neurobiology but also in cancer immunotherapy. Using this technology, it is possible to manipulate the various cellular signaling involved in the programmed cell deaths in cancer treatment. 

Like all emerging technologies, this technique has some limitations to translate in the clinic. As in any gene therapy method involves delivering the sound-sensitive proteins genes in targeted subpopulations cells. Whereas Adeno-associated virus (AAV) vectors are exhibiting good potential in a clinical setting, obstacles such as host immune response, gene transfer, and liver clearance still remain. However, many further investigations are guaranteed to confirm the safety and efficacy of sonogenetics and adjust factors and parameters before clinical practice.

## Ethical approval

This article does not contain any studies with human participants or animals performed by the author.
